# A Speedy and Inexpensive Process for Obtaining Radiographic Reduction on Bromide Paper

**Published:** 1919

**Authors:** 


**A Speedy and Inexpensive Process for Obtaining Radiographic Reduction on Bromide Paper.** By Major BOUGOURD. *Bulletin de Radiographie Militaire*, February 1918.

At the Laboratory of Radiography of the 37th Hospital at Granville a rapid and economical process for obtaining reductions of radiographic plates is used.

In front of a well-lighted window of which the pane has been provided with opaque glass, a wooden frame meant to receive the intermediaries for the different sizes of the plates is placed.

At the proper distance is a small camera, 13 $\times$ 18 centimeters, with rectilinear objective and a focussing plate of ground glass.

After the plate is exposed on the window in its intermediary, the surface protected by black paper masks, the focus is taken according to the size of proofs required. It is rather better to use the diaphragm to get a greater accuracy of details, then the ground-glass plates are replaced by a masked frame holding, instead of sensitive plates, a sheet of rapid bromide paper protected by a glass plate to avoid creasing.

The time of exposure varies according to the intensity of the daylight or the strength of the plate, generally ten seconds for a normal photograph. It only remains to develop carefully. To avoid any blurring it is wise to mask the rest of the window with curtains or blinds.

This speedy and inexpensive process gives very clear reductions, and the quantity of bromide paper used is very small, sizes being 9 $\times$ 12, or 8 $\times$ 10 (centimeters).

The reduced proofs can be added to the papers of a case, and will take the place of encumbering drawings or larger photographs.

They can also be used for collections in albums of small dimensions.

